# Acute Decompensated Heart Failure Secondary to Exogenous Triiodothyronine Use in a Young Non-athlete Weightlifter

**DOI:** 10.7759/cureus.5964

**Published:** 2019-10-22

**Authors:** Ghassan Daher, Ihab Hassanieh, Nikhil Malhotra, Lisa Alderson

**Affiliations:** 1 Internal Medicine, Saint Louis University School of Medicine, St. Louis, USA; 2 Cardiology, Saint Louis University School of Medicine, St. Louis, USA

**Keywords:** heart failure, performance enhancing drugs, triiodothyronine, clenbuterol, cardiogenic shock

## Abstract

Heart failure (HF) is a common cardiovascular disorder and is associated with increased morbidity and mortality. HF is usually detected in the elderly population, in particular, in patients with coronary artery disease, valvular disease, myocarditis, and hypertension. Acute decompensated HF in previously healthy young patients should raise suspicion for other rare etiologies. We report a case of a 28-year-old male presenting with acutely decompensated HF secondary to performance-enhancing drugs (PED). The use of non-regulated, non-approved PED has become a world-wide health problem with patients often unaware of the potentially serious and fatal side effects.

## Introduction

Heart failure (HF) affects more than 26 million people worldwide and 5.1 million people in the United States, with the median age at the time of diagnosis being 75 years. The most common etiologies of HF include ischemic heart disease, dilated (idiopathic) cardiomyopathy, myocarditis, and valvular heart disease [1]. Acute onset HF in a previously healthy young patient should raise the suspicion for rare etiologies. With the recent influence of social media on the upcoming generation, many young males and females are taking extreme measures to achieve what is portrayed as the ideal body image [2]. This has led to the use of various non-approved products that include performance-enhancing drugs (PED) such as anabolic steroids, growth hormones, insulin-like growth factor 1, clenbuterol, amino-acids, whey protein, over-the-counter weight loss pills, and triiodothyronine (T3) containing supplements [3]. Patients are often unaware of the potentially serious side effects and are heavily influenced by promotional advertisements. We report a case of a 28-year-old healthy man presenting to the emergency department with signs and symptoms indicative of acute decompensated HF and respiratory distress.

## Case presentation

**Figure 1:**
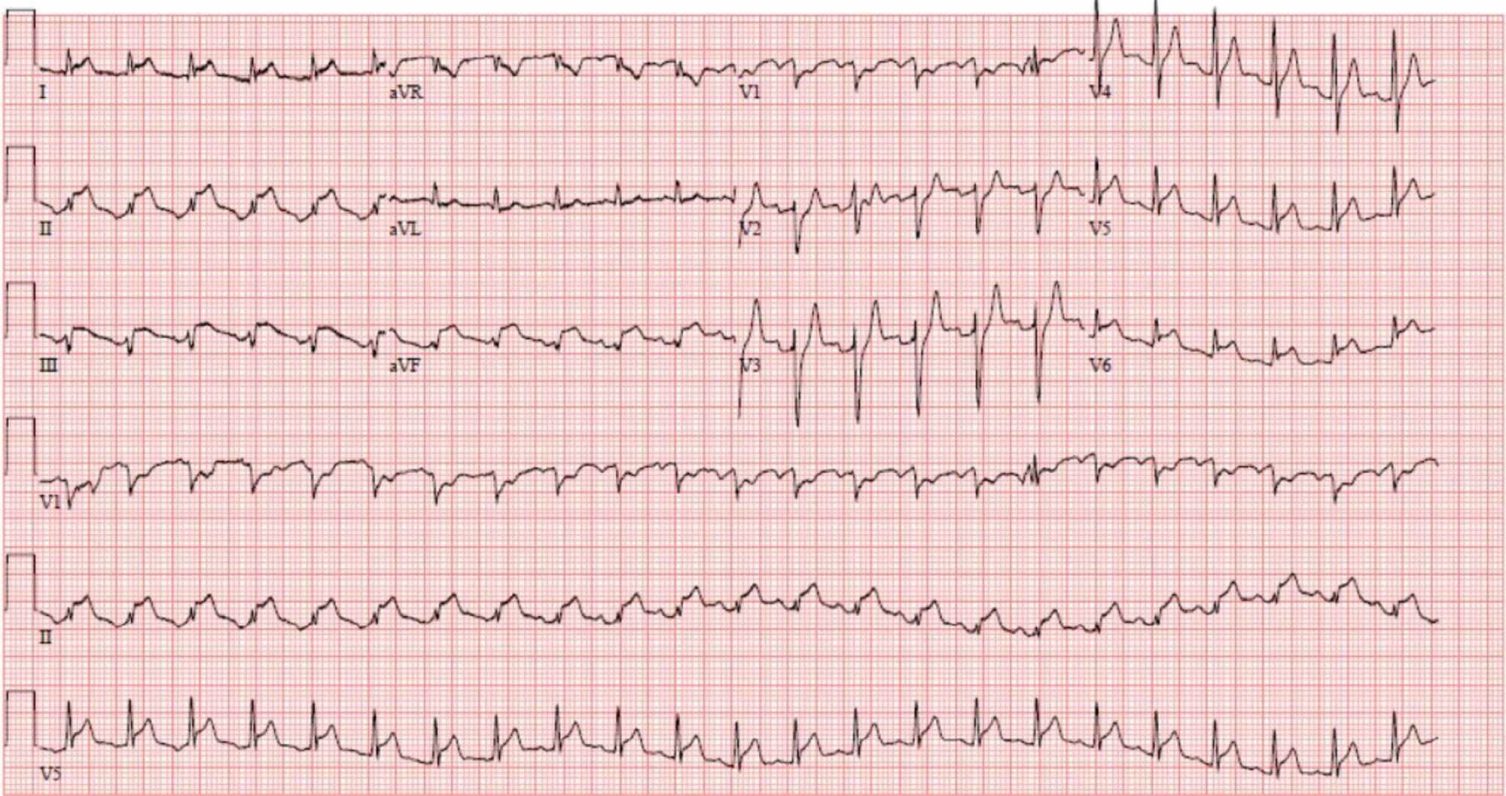
A 12-lead electrocardiogram with ST segment elevation in leads II, III, aVf, V5, and V6

A previously healthy 28-year-old male presented with severe dyspnea and profuse diaphoresis. Initial evaluation and physical exam revealed fever, hypotension, tachycardia, prominent jugular venous distention, bilateral pulmonary crackles, and accessory respiratory muscle use warranting emergent intubation and pressor support. Initial laboratory work-up was remarkable for leukocytosis with a white blood cell (WBC) count of 40, potassium level of 6, creatinine 1.5, lactic acid 0.8, troponin 14.5, mild elevation of the liver enzymes, respiratory acidosis, and a negative urine drug screen. A 12-lead electrocardiogram showed ST-segment elevations in the inferolateral leads (Figure 1). Emergent coronary angiography revealed patent coronaries but noted severe global hypokinesis with an ejection fraction (EF) of 10%. An intra-aortic balloon pump was placed for adjunct hemodynamic support.

Collateral history obtained from the patient’s family revealed that he has been consuming over-the-counter supplements and probable anabolic steroids to enhance his body physique for a summer trip. Additional work-up revealed a negative respiratory viral panel, non-reactive human immunodeficiency virus (HIV) antibody testing, thyroid-stimulating hormone (TSH) level of 0.008 uIU/ml, a free thyroxine (T4) level of 0.26 ng/dl (normal: 1-2.5 ng/dl), a free T3 level of 12.6 pg/ml (normal: 2-4 pg/ml), and a low thyroglobulin level. These findings raised suspicion for surreptitious use of PEDs or supplements containing T3, which led to the development of severe thyrotoxicosis. Nephrology, endocrinology, and toxicology were consulted, and recommended supportive treatment with no role for hemodialysis, plasmapheresis or T3 binding agents. This rationale was based on the non-oliguric state of the patient, the short half-life of T3 and its elimination via renal excretion. Given that the patient was intubated and sedated on initial presentation with resultant stabilization of his hemodynamic status, HF medical therapy (angiotensin-converting enzyme inhibitors (ACEI)/angiotensin II receptor blocker (ARB), beta-blockers, vasodilators) and anti-thyrotoxicosis medications were not initiated. The serum level of T3 decreased rapidly and was accompanied by a simultaneous improvement in the patient’s hemodynamic status, requiring less hemodynamic support with each hour.

**Figure 2:**
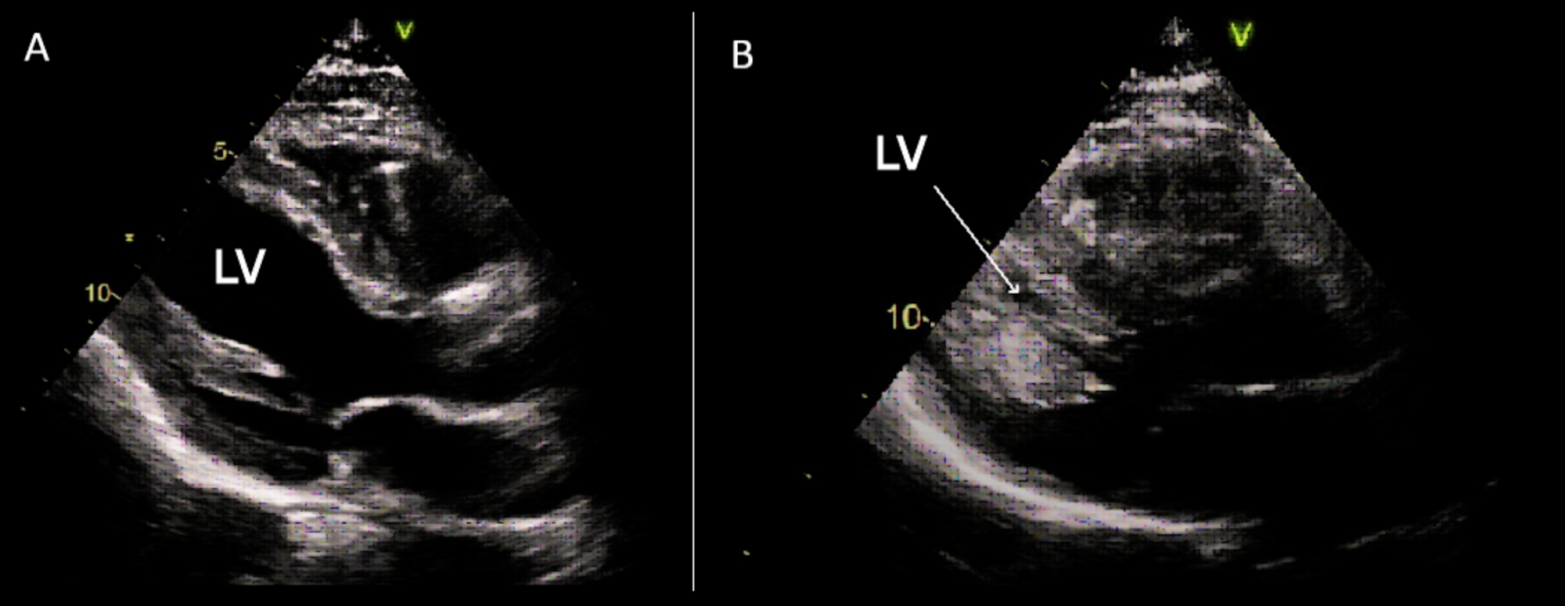
A) Transthoracic echocardiogram performed on day one of hospitalization showing an ejection fraction (EF) of 10%. B) Transthoracic echocardiogram performed on day six of hospitalization showing an EF of 61% LV: Left ventricle

A repeat echocardiography on day two of hospitalization showed an increase in EF to 35%. The patient was ultimately extubated on day three and pressor support was discontinued. On day six of hospitalization, another limited echocardiogram showed a normal EF of 61% with no regional wall motion abnormalities. Figure 2 shows the significant difference in the left ventricular systolic function noted on echocardiography done on day one and day six, respectively.

At this point, the patient was back to his baseline physical health. Upon interviewing the patient after his recovery, he specifically reported taking: clenbuterol 1.5 mg daily, triiodothyronine 75 mcg/day, testosterone enanthate 500 mg weekly, and trenbolone (an anabolic steroid) 400 mg weekly for the past six months.

## Discussion

The use of PED is not only limited to professional athletes but has also expanded to non-athlete weightlifters and young adults in an attempt to achieve the portrayed socially accepted body image [4]. A combination of PED is usually used with no oversight or recommendations from a health-care professional. In this case, our patient was found to have used a combination of anabolic steroids (trenbolone), triiodothyronine, testosterone, and clenbuterol. Given the patient’s rapid clinical improvement in parallel with a decrease in T3 serum levels, normal coronaries, absence of valvular pathology, no history of alcohol use or intravenous drug use, and a negative respiratory viral panel, other etiologies of acute decompensated HF were deemed less likely and no further cardiac evaluation and imaging was pursued. 

The thyroid gland is a major regulator of the body’s basal metabolic rate and cardiovascular hemodynamics. Thyroxine (T4) and to a lesser extent T3 are produced in the thyroid and then released into the peripheral circulation. T4 is then converted by 5’ deiodinase enzyme in the peripheral tissue into the active T3 form which mediates most of the thyroid gland functions. T3 binds to the thyroid hormone receptor (THR) found on the cardiac myocytes and initiates a cascade of intracellular events and leads to direct modulation of membrane ion channels [5]. These include an increased transcription of the alpha myosin gene and sarcoplasmic Ca-ATPase and increased expression of the membrane Na/K exchange pump, β adrenergic receptors, and voltage-gated potassium channels, all of which lead to increased cardiac contractility and heart rate. T3 also has a direct effect on peripheral vasculature. It decreases the arteriolar resistance and thus decreases the systemic vascular resistance. This is sensed by the kidneys which in turn activates the renin-angiotensin system that leads to an increase in the total blood volume and preload [5]. The combined effects of the above-mentioned events lead to an increase in contractility and cardiac output. However, if such hemodynamic state is sustained chronically over a period of several months or years it can paradoxically lead to HF. This has been described by some experts in the literature as high output HF leading to dilated cardiomyopathy or tachycardia-induced HF in patients in which the heart function returned to normal after reversing the thyrotoxic state [6-7]. A proposed mechanism of cardiomyopathy in our patient's case is increased cytosolic calcium due to decreased duration of the action potential and thus less calcium available for myocytic contraction leading to a decrease in EF [7].

Thyroid hormones are regulated by a negative feedback mechanism across the hypothalamic/pituitary/thyroid axis. Therefore, in patients taking exogenous T3, the body will sense a hyperthyroidism state and shut down the secretion of thyrotropin-releasing hormone, TSH, and T4 as seen in our case [8].

In the aforementioned case, the patient’s hypermetabolic state and cardiogenic shock can be attributed to exogenous triiodothyronine intake. It is evident that as the serum level of T3 decreased, the hemodynamic support requirements decreased, and the cardiac function improved simultaneously (Table 1). Given the short half-life of T3 and its high protein binding capacity in circulation; dialysis, plasmapheresis, and binding agents had no therapeutic role [9].

**Table 1:** Evolution of laboratory and echocardiographic findings T3 level on day one at 12.49 which rapidly decreased on subsequent days to 2 on day three and 1 on day six. Ejection fraction on day one at 10% improved to 35% on day three and 61% on day six. T3: Triiodothyronine, T4: Thyroxine, TSH: Thyroid-stimulating hormone.

		Day 1	Day 3	Day 6
T3 (pg/ml)		12.49	2	1
Free T4 (ng/dl)		0.26	0.3	0.4
TSH (uIU/ml)		0.008	0.016	0.017
Ejection Fraction (%)		10	35	61

It is worth mentioning that clenbuterol is an agonist to β-1, β-2 and β-3 receptors [10]. It is not FDA approved for medical use in the United States but has been reportedly used abroad as a bronchodilator in the setting of acute pulmonary exacerbations. β-3 agonism is responsible for enhancing the bodybuilder’s physique by increasing lipolysis and thus decreasing adipose tissue. Clenbuterol’s β-2 agonism has a double-edged effect. It induces the increase in muscle mass but also mediates most of the drug side effects including tachycardia, increased metabolic state, and hypotension. Therefore, toxicity with clenbuterol can mimic and portray septic-like picture leading physicians to treat empirically with intravenous fluids and vasopressors to achieve hemodynamic stability. However, treatment is dramatically different and such early interventions can be detrimental as vasopressors will further promote tachycardia and worsen the current cardiac state. Physicians should be very diligent when faced with such cases and have a wide differential in mind as the treatment of such toxicity is β-adrenergic antagonism. Esmolol, a short-acting easily titratable β-antagonist, is usually preferred [11]. Consequently, clenbuterol and T3 likely potentiated each other’s effect leading to the patient’s near-fatal presentation.

## Conclusions

The use of non-regulated, non-approved PEDs has become a worldwide health problem. Social media has created a false perception of reality and has greatly influenced young adults to use and self-administer PED to enhance their self-esteem and image. Physicians should always consider the possibility of PED abuse in a young, previously healthy bodybuilder presenting with acute cardiogenic shock and multi-organ failure.
